# The Effectiveness of Janus Kinase Inhibitors for the Management of Relapsing Takayasu Arteritis: A Spanish Real-World Study and Comprehensive Review of the Literature

**DOI:** 10.3390/life16061028

**Published:** 2026-06-19

**Authors:** Javier Loricera, Javier Narváez, Susana Romero-Yuste, Valentina Emperiale, Iván Ferraz-Amaro, Carmen Secada-Gómez, Adrián Martín-Gutiérrez, Ricardo Blanco

**Affiliations:** 1 Department of Rheumatology, Hospital Universitario Marqués de Valdecilla, 39008 Santander, Spain; carmensecada@hotmail.com (C.S.-G.); adrianmartingutierrez@gmail.com (A.M.-G.); rblancovela@gmail.com (R.B.); 2 Immunopathology Group, Instituto de Investigación Valdecilla (IDIVAL), 39011 Santander, Spain; 3 Department of Medicine and Psychiatry, University of Cantabria, 39011 Santander, Spain; 4 Department of Rheumatology, Hospital de Bellvitge, 08907 Barcelona, Spain; fjnarvaez@bellvitgehospital.cat; 5 Department of Rheumatology, Complejo Hospitalario Universitario de Pontevedra, 36161 Pontevedra, Spain; susanaromeroyuste@gmail.com; 6 Department of Rheumatology, Hospital Universitario Príncipe de Asturias, 28805 Alcalá de Henares, Spain; vemperiale@gmail.com; 7 Department of Rheumatology, Complejo Hospitalario Universitario de Canarias, 38320 Tenerife, Spain; iferrazamaro@hotmail.com

**Keywords:** Takayasu arteritis, large-vessel vasculitis, Janus kinase inhibitors, baricitinib, tofacitinib, upadacitinib

## Abstract

**Background**: A significant proportion of individuals with Takayasu arteritis (TA) experience relapses notwithstanding standard treatment with glucocorticoids, and conventional synthetic or biologic disease-modifying antirheumatic drugs (DMARDs). As the Janus kinase/signal transducer and activator of transcription (JAK/STAT) signaling pathway contributes to the pathogenesis of TA, JAK inhibitors (JAKi) could represent a viable therapeutic alternative. This study assessed the effectiveness of JAKi in patients with relapsing TA within a real-world setting in a country with a low incidence of TA such as Spain and included a comprehensive review of the literature. **Methods**: we conducted a retrospective analysis of TA patients managed with JAKi for recurrent disease across three Spanish centers. Evaluated outcomes comprised clinical remission, clinical and analytical remission, glucocorticoid-sparing effect, improvement in imaging techniques, and adverse events. A systematic literature search was performed to identify further cases of TA treated with JAKi. **Results**: six patients (83.3% females) with a mean age 48.5 years and relapsing TA received JAKi therapy: baricitinib (*n* = 2); tofacitinib (*n* = 2), and upadacitinib (*n* = 2). Before JAKi therapy, all (100%) patients had received conventional synthetic immunosuppressants, and four (66.7%) biologics. Clinical remission was achieved in 2/6 (33.3%), 3/5 (60%), 3/5 (60%), 2/3 (66.7%), and 2/2 (100%) patients at 1, 3, 6, 12 and 18 months, respectively. Clinical + analytical remission was observed in 1/6 (16.7%), 2/5 (40%), 2/5 (40%), 2/3 (66.7%), and 2/2 (100%) patients, respectively. Two patients who underwent a follow-up PET/CT imaging showed partial improvement in both. After a median (IQR) follow-up of 9.5 (6.0–16.7) months, one (16.7%) patient discontinued the initial JAKi due to no improvement and one patient discontinued it because was diagnosed with tonsillar neoplasia. The literature search identified another 166 JAKi-treated TA cases with clinical improvement reported for the majority of them. **Conclusions**: this real-world analysis and literature review suggest that JAKi could be effective in the management of TA, including for those patients who have failed established glucocorticoid-sparing strategies.

## 1. Introduction

Takayasu arteritis (TA) is a form of large-vessel vasculitis characterized by chronic granulomatous inflammation that primarily affects the aorta and its major branches [1,2,3]. Although it is most frequently observed in young women in East Asia, Africa, and South America, epidemiological data from Europe indicate an annual incidence of 0.4 to 1.5 per million and a prevalence ranging between 4.7 and 33 per million [4,5,6,7,8].

Due to its association with substantial morbidity, the clinical management of TA remains a significant challenge.

Glucocorticoids (GC) are the primary therapeutic approach; however, treatment failure and relapses occur frequently. Consequently, the introduction of conventional synthetic disease-modifying antirheumatic drugs (DMARDs), including methotrexate (MTX), leflunomide (LFN), azathioprine (AZA), cyclophosphamide (CYP), and mycophenolate mofetil (MMF), is often necessary to achieve clinical remission. Nevertheless, the effectiveness of these agents is constrained by inconsistent efficacy and high incidence of severe side effects [9,10,11].

Given that TNF-α and interleukin (IL)-6 are key components in the pathogenesis of TA, research has focused on their targeted inhibition. Studies investigating TNF-α and IL-6 receptor (IL-6R) blockade in patients with refractory TA have yielded varied results [12,13,14,15,16,17]. The ACT-Bridge study, which assessed a 52-month course of subcutaneous tocilizumab (TCZ) in a Japanese cohort, reported a 20% relapse rate [18]. Therefore, other treatment options are greatly needed for patients with TA.

In recent years, the pivotal role of the Janus kinase/signal transducers and activators of transcription (JAK/STAT) signaling pathway in immune-mediated conditions has been exploited through the development of Janus kinase inhibitors (JAKi). These small molecules function by blocking the activity of type I and II cytokines [19]. In patients with TA, there is a marked upregulation of JAK/STAT pathways, interferons, and various cytokine-related genes within CD4+ and CD8+ T cells [20]. JAK inhibition effectively suppresses key citokines involved in the pathogenesis of TA, such as IL-6, IL-12, IL-17,IL-23, and both type 1 and 2 interferons [21]. Moreover, these inhibitors also modulate the activity of macrophages and natural killer cells, which are implicated in the progression of the disease [22].

At present, published evidence regarding the efficacy of JAKi in TA is limited, consisting of case reports and a small series of patients [23,24,25,26,27,28,29,30,31,32,33,34,35,36,37,38,39,40,41,42,43,44,45]. Therefore, additional data on this topic would be valuable.

We have retrospectively evaluated the outcomes of the first Spanish series of patients with relapsing TA managed with JAKi in a real-world setting, complemented by a systematic review of the existing literature.

## 2. Patients and Methods

### 2.1. Study Design and Patient Population

We conducted an observational, retrospective analysis of patients diagnosed with TA who received JAKi treatment across three specialist centers in Spain. All patients fulfilled the 1990 American College of Rheumatology classification criteria for TA [46], and/or Sharma criteria [47]. In every instance, vascular involvement was confirmed through at least imaging technique, including 18F-fluorodeoxyglucose positron emission tomography/computed tomography (18F-FDG PET/CT), computed tomography angiography (CT-A), Doppler ultrasound (US), and angiography.

JAKi therapy was initiated at the discretion of the attending rheumatologist for patients experiencing disease relapse despite the use of GC and other immunosuppressive agents, including conventional synthetic and biologic DMARDs. Because this study utilized real-world data, there were no pre-established criteria for the selection of the JAKi, dosage, or the subsequent GC tapering scheme. Factors influencing providers when making those decisions may have included a patient’s preference, provider’s experience and judgment, insurance authorization, cost, and safety.

The study was reported in accordance with STROBE guidelines [48].

### 2.2. Study Assessments and Outcomes

Effectiveness and safety outcomes were evaluated through a systematic review of rheumatology clinical notes, laboratory parameters, and vascular imaging results from medical records. During follow-up, patients were seen by the rheumatology providers at variable intervals, but mostly every one to six months. Data extraction followed a rigorous protocol, and all entries were double-checked to ensure accuracy.

Clinical remission was defined as the total resolution of existing TA symptoms and the absence of new manifestations. Clinical and analytical remission was defined as clinical remission alongside the normalization of the erythrocyte sedimentation rate (ESR) and C-reactive protein (CRP). Relapse was defined as the recurrence of signs or symptoms of TA after at least six months of remission.

Limb claudication was defined by the presence of pain, heaviness, and/or cramping in the extremities. Constitutional syndrome encompassed asthenia, anorexia, and weight loss greater than 5% of the normal body weight over the last 6 months. Fever was defined as a temperature ≥ 38 °C. Blood pressure difference between upper limbs was considered if there was a difference in systolic blood pressure ≥ 20 mm Hg between arms. Headache was present if head pain was of recent development or had different characteristics than usual. Visual manifestations included blurred vision, diplopia, amaurosis fugax, hemianopsia, and permanent unilateral or bilateral blindness.

The ESR was considered to be increased when it was greater than 20 or 25 mm/hour for men or women, respectively. A serum CRP value higher than 0.5 mg/dL was considered abnormal. Anemia was defined as a hemoglobin level ≤ 11 g/dL. Other hematological abnormalities included leukopenia (<4000 leukocytes/µL), lymphopenia (<1500 lymphocytes/µL), neutropenia (<1500 neutrophils/µL), and thrombocytopenia (<100,000 platelets/µL).

Follow-up imaging was conducted at the discretion of the treating physician. To ensure clinical reliability despite the lack of a standardized scoring system (such as the PETVAS), all images were evaluated qualitatively by specialist radiologists or nuclear medicine experts at each referral center. Imaging vascular improvement was defined as the partial or complete improvement of vessel wall thickness, stenosis, or occlusions, along with the absence of new vascular lesions at the follow-up imaging technique as compared with baseline.

### 2.3. Statistical Analysis

Continuous variables were described as median [interquartile range] at each study visit. Longitudinal changes in prednisone dose, erythrocyte sedimentation rate, and C-reactive protein were assessed by comparing each follow-up time point with baseline using the paired Wilcoxon signed-rank test. Only paired observations with available data at both time points were included in each analysis. All tests were two-tailed, and statistical significance was set at *p* < 0.05. Owing to the small sample size and the exploratory nature of the study, findings were considered descriptive and interpreted cautiously. The analysis was conducted using Stata software, version 17/BE (StataCorp, College Station, TX, USA).

### 2.4. Ethical Considerations

The present study was approved by the Cantabria Clinical Research Ethics Committee (internal code 2021.414), and was conducted in accordance with the Declaration of Helsinki and the International Conference for Harmonization. All data extracted from the medical records were stored in a de-identified format prior to the analysis to ensure patient confidentiality. According with the Clinical Research Ethics Committee’s guidelines for retrospective research, informed consent was not required.

### 2.5. Literature Review

We performed a national multicenter retrospective study of patients with refractory TA treated with JAKi in routine clinical practice. A systematic literature search was conducted across PubMed, Embase, and the Cochrane Library from inception to 31 January 2026, to identify all published cases and series of TA treated with JAKi. The search strategy employed a combination of the following keywords: “Takayasu arteritis”, “baricitinib”, “tofacitinib”, and “upadacitinib”. We included all published case reports and series involving human subjects with TA treated with JAKi. Records were excluded if the required clinical or outcome data were not available in the published text. This review was carried out in accordance with the 2020 Preferred Reporting Items for Systematic Reviews and Meta-Analyses (PRISMA) guidelines [49].

## 3. Results

### 3.1. Baseline General Characteristics at JAKi Initiation

A total of six patients (five women and one man) with TA who received treatment with JAKi were included [Table 1]. All patients met both Sharma criteria and 1990 ACR criteria. The imaging techniques performed were as follows: 18F-FDG PET/CT (*n* = 3; 50%), CT-A (*n* = 1; 16.7%), US (*n* = 1; 16.7%), and angiography (*n* = 1; 16.7%). The affected vessels were as follows: ascending thoracic aorta (*n* = 4; 66.7%), aortic arch (*n* = 4; 66.7%), supra-aortic trunks (*n* = 3; 50%), descending thoracic aorta (*n* = 4; 66.7%), abdominal aorta (*n* = 3; 50%), carotid arteries (*n* = 1; 16.7%), vertebral arteries (*n* = 1; 16.7%), subclavian arteries (*n* = 2; 33.3%), brachial arteries (*n* = 1; 16.7%), coronary arteries (*n* = 1; 16.7%), and left iliac artery (*n* = 1; 16.7%).

Five patients had a stenosis and one patient had a 42 mm dilation of the ascending aorta. According to the classification by Numano et al. [50], the types of TA in patients were as follows: type I (*n* = 2; 33.3%), type IIb (*n* = 1; 16.7%), and type V (*n* = 3; 50%). The mean ± SD age at JAKi therapy initiation was 48.5 ± 9.6 years. Overall, two (33.3%) patients received baricitinib (4 mg daily), two (33.3%) tofacitinib (5 mg twice per day), and two (33.3%) upadacitinib (15 mg daily). The median [IQR] time from TA diagnosis to JAKi therapy onset was 29.5 [13.0–51.2] months. Without considering concomitant GC use, JAKi was prescribed as monotherapy in two (33.3%) patients and combined with conventional synthetic immunosuppressive agents in four patients: MTX (*n* = 3; 50%) and MMF (*n* = 1; 16.7%).

The main clinical symptoms and signs of the TA patients at the time of JAKi initiation are described in Table 1. Those included asthenia (*n* = 6; 100%), constitutional syndrome (*n* = 2; 33.3%), upper limb claudication (*n* = 3; 50%), blood pressure differences between upper limbs (*n* = 4; 66.7%), vascular murmurs (*n* = 1; 16.7%), headache (*n* = 1; 16.7%), chest pain (*n* = 2; 33.3%), neck pain (*n* = 2; 33.3%), nausea (*n* = 1; 16.7%), dizziness (*n* = 1; 16.7%). The median [IQR] baseline serum CRP and ESR values were 2.0 [1.2–3.8] mg/dL and 34.0 [24.7–51.5] mm/1st hour. All patients were receiving prednisone at JAKi initiation. The median [IQR] baseline prednisone dose was 10.0 [5.0–22.5] mg/day.

Before JAKi therapy, all (100%) patients had received several conventional synthetic DMARDs such as MTX (*n* = 6; 100%), AZA (*n* = 1; 16.7%), MMF (*n* = 2; 33.3%), and CYP (*n* = 1; 16.7%) [Table 1]. In addition, four (66.7%) patients had been treated with biologics including etanercept (*n* = 1; 16.7%), infliximab (*n* = 4; 66.7%), tocilizumab (*n* = 4; 66.7%), rituximab (*n* = 1; 16.7%), and ustekinumab (*n* = 1; 16.7%) [Table 1].

### 3.2. Clinical Outcomes

**Figure 1:**
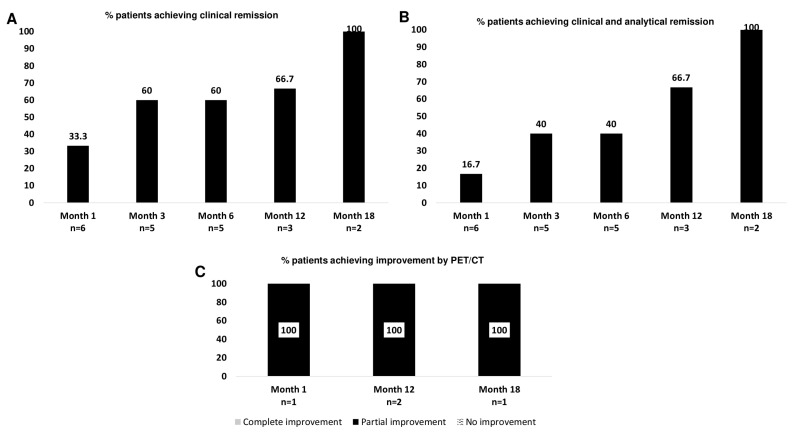
Clinical outcomes of six patients with Takayasu arteritis after JAK inhibitor initiation. Legend: (**A**) Clinical remission; (**B**) Clinical and analytical remission; and (**C**) PET/CT improvement. JAK: Janus kinase, PET/CT: positron emission tomography/computed tomography.

Once on JAKi, patients were followed for a median [IQR] period of 9.5 [6–16.7] months with six patients followed for at least one month, five patients followed for at least three months, five patients followed for at least six months, three patients followed for at least twelve months, and two patients followed for at least eighteen months. Most patients experienced an improvement in clinical symptoms, as well as laboratory parameters throughout treatment with JAKi. Clinical remission was observed at one, three, six, twelve, and eighteen months in 2/6 (33.3%), 3/5 (60%), 3/5 (60%), 2/3 (66.7%), and 2/2 (100%) patients, respectively [Figure 1A]. Clinical and analytical remission was observed at one, three, six, twelve, and eighteen months in 1/6 (16.7%), 2/5 (40%), 2/5 (40%), 2/3 (66.7%), and 2/2 (100%) patients, respectively [Figure 1B].

A patient undergoing treatment with baricitinib underwent ^18^F-FDG PET/CT scan 1 month, 12 months, and 24 months after starting JAKi therapy, showing partial improvement. Another patient receiving tofacitinib underwent a follow-up ^18^F-FDG PET/CT scan 12 months after starting JAKi treatment, showing partial improvement.

The median [IQR] ESR declined from 34.0 [24.7–51.5] mm/1st hour at baseline to 13 [6.7–33.5] mm/1st hour at last follow-up (*p* = 0.094). The median [IQR] serum CRP value decreased from 2.0 [1.2–3.8] at baseline to 0.5 (0.5–1.3) mg/dL at last follow up (*p* = 0.062).

The median [IQR] daily dose of prednisone decreased from 10.0 [5.0–22.5] at baseline to 5.0 [3.1–8.7] mg at last follow up (*p* = 0.125). No patient discontinued GC.

**Figure 2:**
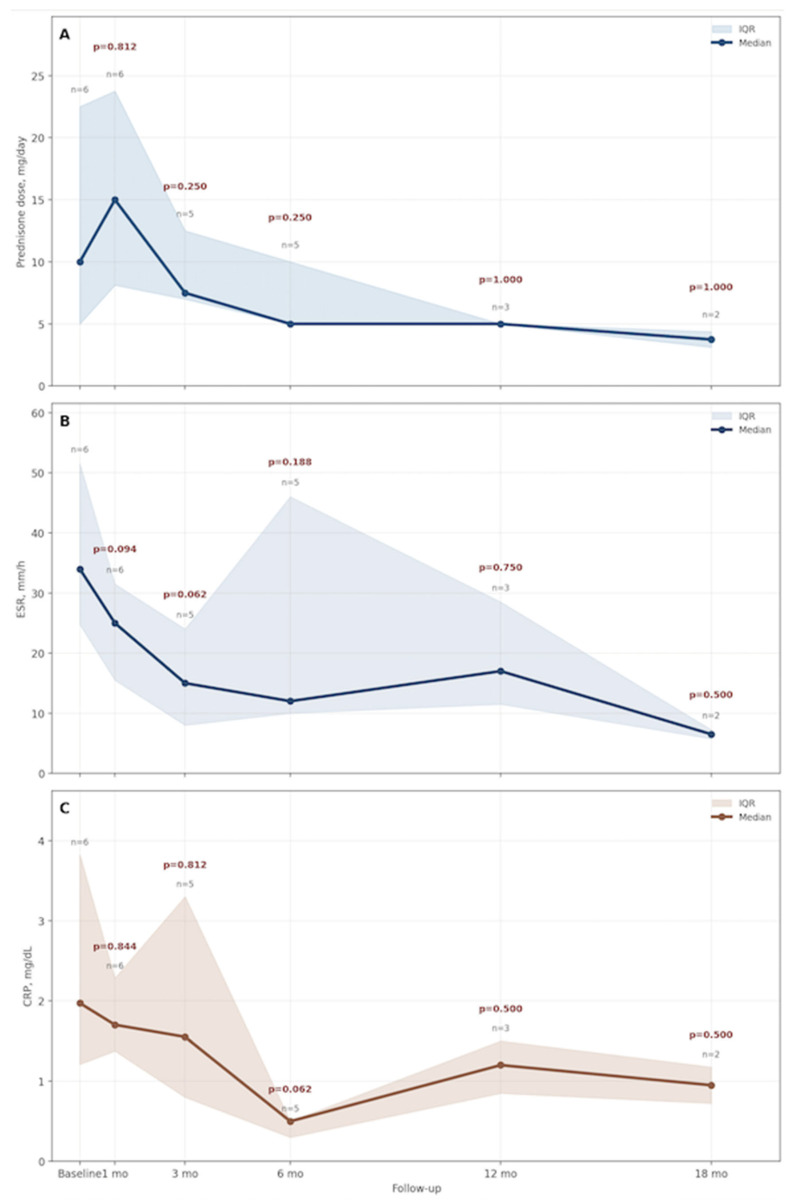
Longitudinal evolution of (**A**) prednisone dose, (**B**) erythrocyte sedimentation rate (ESR), and (**C**) C-reactive protein (CRP) during follow-up. Legend: the solid line represents the median at each visit and the shaded area the interquartile range. Numbers above each time point indicate the number of patients with available data, and *p* values correspond to paired Wilcoxon signed-rank tests versus baseline.

Overall, one (16.7%) patient discontinued JAKi therapy due to persistence of active disease with baricitinib. Figure 2 illustrates the evolution of CRP, ESR and prednisone dosage during follow-up.

### 3.3. Safety

One patient on tofacitinib developed a tonsillar neoplasia requiring permanent JAKi discontinuation. No cases of thromboembolism, serious adverse cardiovascular events, or significant cytopenias were observed during follow-up.

### 3.4. Literature Review

**Figure 3:**
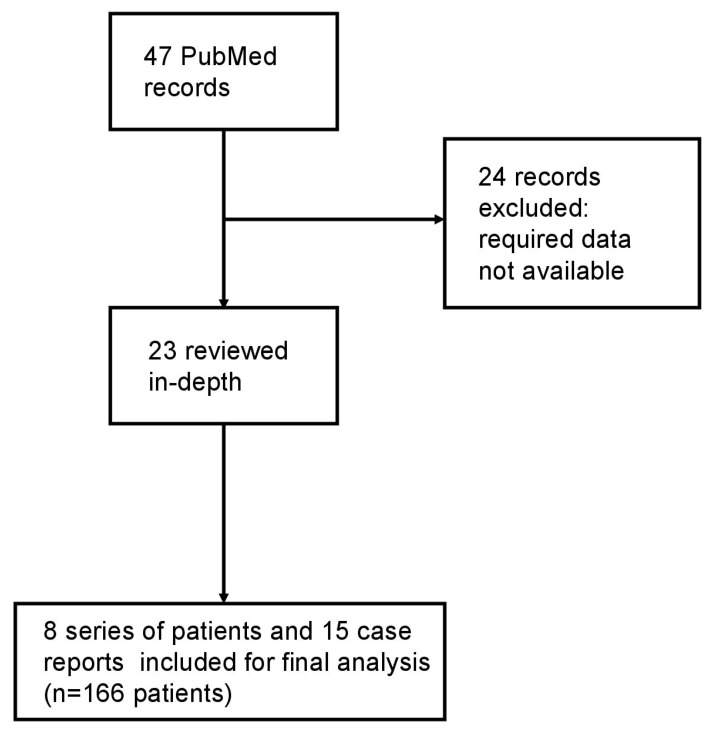
Flow chart of patient selection from the literature review.

Our literature review identified 15 case reports and 8 series of patients with a total of 166 Takayasu arteritis patients treated with JAKi: tofacitinib (*n* = 128; 77.1%), baricitinib (*n* = 26; 15.7%), upadacitinib (*n* = 9; 5.4%), and ruxolitinib (*n* = 2; 1.2%) [Figure 3 and Table 2]. Most patients showed clinical improvement, and adverse events were infrequent. Herpes zoster was the most commonly reported event [Table 2].

## 4. Discussion

The results from this observational study and the literature review suggest that JAKi may be effective in TA patients, even in cases where conventional synthetic and biologic DMARDs have previously failed. After a median follow-up of 9.5 [6–16.7] months, a significant proportion of the patients receiving JAKi in our series experienced clinical and analytical improvement. In addition, the patients in our cohort were able to significantly reduce their daily prednisone doses to a median of 5 mg.

GC have been the cornerstone of the treatment of TA for decades. Nonetheless, relapses are common when their dosage is gradually tapered. Between 46 and 84% of patients with TA require a second drug to achieve remission and successfully taper GC [51]. For this reason, other drugs, such as MTX, LFN, AZA, CYP, and MMF (11), as well as TNF-α and/or IL-6R inhibitors, are used [12,13,14,15,16,17]. Indeed, TNF-α and IL-6R inhibitors may be used in inducing and maintaining remission. However, results are still controversial, and head-to-head RCTs are needed [9,12,13,14,15,16,17]. Several cohort studies on the successful use of different TNF-α inhibitors have been reported in patients with TA, but no RCTs have been published. In a meta-analysis in which 19 observational studies on TNF-α inhibitors in TA were assessed, the relapse rate was estimated as 32% [52]. The effectiveness of TCZ in TA has been reported in several cohort studies [9,53,54]. Besides observational studies, the phase 3 RCT TAKT study was published in 2018. In this study, 36 patients with relapsing TA were randomized to receive TCZ 162 mg subcutaneous weekly or placebo. Nonetheless, the primary endpoint was not met [17]. In the longer-term open-label extension of this trial, 17.9% and 67.9% of patients showed improvement and stabilization, respectively, on imaging techniques after 96 weeks, while four patients showed progression of vascular involvement [55].

The activation of the JAK/STAT signaling pathway is intimately associated with the production of numerous cytokines, notably type I and II IFN, which are integral to the pathogenesis of various autoimmune conditions, including vasculitis [56,57]. Within the context of TA, this pathway performs a critical role; specifically, Th1 and Th17 cells are primary mediators in the disease development. Th1 cells are linked to the activity of STAT1, STAT2, and STAT4, whilst Th17 cells are associated with STAT3. [56]. Supporting this, transcriptomic analyses of CD4+ and CD8+ T cells in large TA cohorts have revealed heightened expression of genes within the JAK/STAT axis, including IL-12, IL-17, IL-19, IL-22, and both type I and II IFNs [37].

Clinical data on the efficacy and safety of JAKi in TA are still scarce and limited to a few case reports or small case series [Table 2] [23,24,25,26,27,28,29,30,31,32,33,34,35,36,37,38,39,40,41,42,43,44,45].

Our clinical results demonstrated clinical remission in 60% of patients at six months and 100% in those followed to eighteen months.

In comparison with the literature, which predominantly focuses on tofacitinib, our cohort provides valuable insights into the use of baricitinib and upadacitinib.

Although the most appropriate approach for assessing disease progression would have been to use validated disease activity scores, such as ITAS2010 or DEI.Tak, due to this was a retrospective analysis of real-world clinical practice across three different centers in Spain, these specific scoring tools were not systematically recorded in patients’ medical records at each visit. Consequently, it was not possible to calculate them accurately for all patients during the follow-up period.

Wang et al. [41] compared the efficacy and safety of LFN and tofacitinib in a prospective study of 67 TA patients during a period of 12 months. Thirty-five patients were treated with LFN and thirty-two with tofacitinib. The proportion of patients with persistent remission from the 6th to 12th months and GC doses equal to or less than 7.5 mg/day at 12 months was higher in the patients with tofacitinib, observed in 46.9% of the tofacitinib group. Relapses were observed in 17.1% of patients with tofacitinib. Twenty-five percent of the tofacitinib group showed imaging improvement.

Kong et al. [39] compared the efficacy and safety of tofacitinib and MTX in a prospective TA cohort in China of 53 patients with TA. Twenty-seven patients received tofacitinib and twenty-six patients MTX for remission induction. Tofacitinib led to a higher complete remission rate at months 6 and 12 (85% vs. 61%, *p* = 0.07; and 88% vs. 56%, *p* = 0.02, respectively), with a longer median relapse-free duration and a similar adverse event rate.

Our findings regarding baricitinib complement recent Chinese data. Zhou et al. [42] conducted a prospective study in a national tertiary referral center for TA in China with baricitinib 4 mg daily for a median follow-up of 15.3 months in 10 patients with refractory TA. After 6 months, 60% of patients had an overall treatment response. During follow-up, 40% of patients maintained overall treatment response. Eight patients tapered or maintained the same dose of GCs, and two patients were able to withdraw GCs, while one patient had to stop baricitinib owing to liver dysfunction.

Furthermore, recent trial data demonstrated an 80% overall response rate at 24 weeks specifically in patients’ refractory to TNF-α inhibitors [43], supporting our observation that JAKi can salvage patients who have failed multiple biologic lines.

Similarly, our successful use of upadacitinib mirrors emerging case reports showing its benefit in refractory pediatric cases and patients with concomitant spondyloarthritis [30,31].

While our overall clinical response rate at six months (60%) appears lower than some reported tofacitinib cohorts (up to 87–88%), this likely reflects the high degree of treatment refractoriness in our Spanish cohort, where all patients failed multiple conventional synthetic immunosuppressants and 66.7% failed prior biologics.

A pivotal advantage of JAKi identified in both our series and the literature is their potent GC-sparing effect. Although our series did not achieve statistical significance in the reduction of prednisone dosage throughout follow-up, probably due to the small number of patients, a clear trend toward dosage reduction was observed, with a reduction from 10 mg to 5 mg. This is consistent, for example, with the cohort published by Wang et al. [41], where tofacitinib achieved a significantly higher prevalence of low-dose GC remission (≤7.5 mg/day) compared to LFN (46.9% vs. 17.1%). This ability to maintain remission while minimizing long-term GC toxicity is crucial for a disease typically affecting young women.

With regard to imaging findings, only two patients of our series had follow-up PET/CT, and both showed only partial improvement, which shows that complete resolution of vascular inflammation in TA is very difficult to achieve and requires longer periods of treatment. Similarly, in the cohort of patients published by Wang et al., vascular improvement was only found in 28.1% of patients treated with tofacitinib [41].

The safety profile of JAKi in our study was generally consistent with the literature. One patient treated with tofacitinib for 13 months was diagnosed with a tonsillar neoplasia, leading to permanent treatment discontinuation. Whilst a definitive causal relationship cannot be established in this individual case, the association between JAKi and an increased risk of certain malignancies (including solid tumors and lymphomas) has been a subject of ongoing evaluation. Most published data report infections, particularly herpes zoster, as the most frequent adverse event [Table 2]. We entirely acknowledge that our median follow-up duration of 9.5 months is relatively short for a robust evaluation of long-term safety outcomes, and, although no major adverse cardiovascular events or thromboembolic episodes were observed, given the intrinsic vascular nature of TA, close and prolonged monitoring remains essential for patients receiving these therapies.

Because the current evidence regarding the utility of JAKi in TA is almost entirely derived from case reports and small uncontrolled series, there is an inherent tendency for successful outcomes to be over-reported, whilst treatment failures may remain unpublished. We agree that this context is essential for the balanced interpretation of our findings.

The main limitations of our study are its retrospective nature, which could have introduced bias due to missing data, and the small sample size. In addition, incomplete documentation of data related to individual prednisone tapering courses made the calculation of cumulative prednisone dose, a key outcome measure in TA, inaccurate and therefore not analyzable. The presence of concomitant medications is a limitation that prevents us from definitively attributing the clinical improvement to JAKi alone. On the other hand, the lack of standardized activity scores may introduce a degree of subjectivity to the definition of clinical remission. The non-standardized and non-systematic nature of the imaging evaluation weakens the effectiveness analysis.

Despite these limitations, information about key efficacy and safety events (e.g., remission, relapse, serious adverse events, and drug discontinuation) was unequivocally present in the data source, which makes our estimations reliable. Moreover, to our knowledge, this is the first study to date evaluating outcomes of TA patients treated with JAKi in Spain.

To provide broader context for our small cohort, we have further contextualized our results within the literature review of 166 patients, which helps to mitigate the limitations of our local series.

## 5. Conclusions

In summary, our findings are preliminary real-world observations that suggest that baricitinib, tofacitinib, and upadacitinib may serve as effective therapeutic options for refractory TA. These JAKi appear to offer a GC-sparing effect and are capable of inducing remission in patients who have not responded to multiple conventional synthetic and biologic DMARDs therapies. Although these results are promising, the limited size of our patient cohort and the variability observed in the existing literature highlight a pressing requirement for large-scale, randomized controlled trials. Such studies are essential to definitively determine the comparative effectiveness and long-term safety profiles of various JAKi within this specific form of vasculitis.

## Figures and Tables

**Table 1:** Main features before and after the initiation of JAKi therapy in the six patients with refractory Takayasu arteritis.

Case	Age/Sex	Numano Classification	JAKi	Previous Immunosuppressive Drugs	Clinical Symptoms and Signs at JAKi Initiation	CRP (mg/dL)/ESR (mm/1 h) at JAKi Initiation	CRP (mg/dL)/ESR (mm/1 h) at Last Visit	Prednisone Dose at JAKi Initiation (mg/Day)	Prednisone Dose at Last Visit (mg/Day)	Imaging Technique Before JAKi Initiation	Imaging Technique After JAKi Initiation (Months)	Follow-Up with JAKi (Months)	Outcome	Adverse Events
1	30/F	IIb	Baricitinib	MTX, AZA, MMF, ETN, IFX, TCZ, RTX, USTE	Asthenia, upper limb claudication, blood pressure differences between limbs	4.3/30	0.2/4	5	2.5	CT-A, US: aortic arch, supraaortic trunks, ascending and descending thoracic aorta, carotid arteries, vertebral arteries	PET/CT (1, 12 and 18 months): partial improvement	24	Complete clinical and analytical improvement	None
2	57/M	V	Tofacitinib	MTX, IFX, TCZ	Asthenia, constitutional syndrome	0.6/7	0.5/5	5	2.5	PET/CT: ascending and descending thoracic aorta, aortic arch, abdominal aorta, subclavian arteries, brachial arteries	PET/CT (12 months): partial improvement	18	Complete clinical and analytical improvement	None
3	51/F	I	Tofacitinib	MTX	Asthenia, upper limb claudication, blood pressure differences between limbs, neck pain	1.1/38	1.2/40	15	5	Angiography: supraaortic trunks	Not performed	13	Complete clinical improvement	Tonsilar neoplasia
4	49/F	I	Baricitinib	MTX	Asthenia, constitutional syndrome, upper limb claudication, blood pressure differences between limbs, chest pain, neck pain	2.4/56	0.5/12	30	30	CT-A: aortic arch, left subclavian artery	Not performed	6	No improvement	None
5	55/F	V	Upadacitinib	MTX, CYP, IFX, TCZ	Asthenia, blood pressure differences between limbs, vascular murmur, headache, nausea, dizziness	1.5/68	0.1/46	25	10	PET/CT, CT-A: ascending and descending thoracic aorta, supraaortic trunks, abdominal aorta, left iliac artery	Not performed	6	Clinical improvement	None
6	49/F	V	Upadacitinib	MTX, MMF, IFX, TCZ	Asthenia, chest pain	5.7/23	1.3/14	5	5	PET/CT, CT-A: ascending and descending thoracic aorta, aortic arch, abdominal aorta, coronary arteries	Not performed	2	No improvement	None

**Abbreviatures:** AZA: azathioprine, CRP: C-reactive protein, CT-A: computed tomography angiography, CYP: cyclophosphamide, ESR: erythrocyte sedimentation rate, ETN: etanercept, F: female, IFX: infliximab, JAKi: Janus kinase inhibitor, M: male, MMF: mycophenolate mofetil, MTX: methotrexate, PET/CT: positron emission tomography/computed tomography, RTX: rituximab, TCZ: tocilizumab, US: ultrasound, USTE: ustekinumab.

**Table 2:** Literature review of patients with Takayasu arteritis treated with Janus kinase inhibitors.

Reference	Cases	Sex	Age, Mean ± SD or Median [IQR]	JAKi	Previous cDMARDs	Previous bDMARDs	Concomitant Medication (Apart from Glucocorticoids)	Follow-Up (Months), Mean ± SD or Median (Range)	Outcome	Adverse Events
Kuwabara et al., 2019 [23]	1	Female	32	Tofacitinib	None	Adalimumab, tocilizumab	None	3	Clinical improvement	None
Ríos-Rodríguez et al., 2020 [24]	1	Male	38	Tofacitinib	Methotrexate, sulfasalazine	Etanercept, infliximab, certolizumab pegol, secukinumab	Methotrexate	12	Clinical improvement	None
Sato et al., 2020 [25]	1	Female	17	Tofacitinib	Azathioprine	Golimumab	Mesalazine (for ulcerative colitis)	6	Clinical improvement	None
Yamamura et al., 2019 [26]	1	Male	26	Tofacitinib	Azathioprine, cyclosporine	Infliximab, tocilizumab	Methotrexate	12	Clinical improvement	None
Wang et al., 2022 [27]	1	Male	21	Tofacitinib	Methotrexate, azathioprine	None	None	9	Clinical improvement	None
Bhowmick K et al., 2023 [28]	1	Female	22	Tofacitinib	Methotrexate	Tocilizumab	None	12	Clinical improvement	None
Ru C et al., 2023 [29]	1	Male	36	Tofacitinib	Sulfasalazine, methotrexate	None	None	6	Clinical improvement	None
Pfeil A et al., 2025 [30]	1	Female	24	Upadacitinib	Sulfasalazine	Adalimumab, certolizumab	None	4	Clinical improvement	None
Liang B et al., 2025 [31]	1	Female	10	Upadacitinib	Methotrexate, cyclophosphamide	Tocilizumab	None	10	Clinical improvement	None
Callejas JL et al., 2026 [32]	1	Female	37	Upadacitinib	None	Tocilizumab, adalimumab	Adalimumab	No data	Clinical improvement	None
Sulu B et al., 2025 [33]	1	Female	17	Upadacitinib	Cyclophosphamide	Adalimumab, infliximab	None	12	No improvement	None
Belfeki N et al., 2024 [34]	1	Female	33	Upadacitinib	Methotrexate	Tocilizumab, infliximab	Methotrexate, infliximab	12	Clinical improvement	Two infections
Palermo et al., 2020 [35]	2	Female (*n* = 2)	16 ± 2.8	Tofacitinib (*n* = 2)	Methotrexate (*n* = 2), azathioprine (*n* = 1), mycophenolate mofetil (*n* = 1)	Adalimumab (*n* = 2), infliximab (*n* = 1), rituximab (*n* = 2), tocilizumab (*n* = 2)	Mycophenolate mofetil (*n* = 1)	4.5 ± 3.5	No improvement (*n* = 2)	None
Ino et al., 2022 [36]	2	Female (*n* = 1), male (*n* = 1)	22.5 ± 4.9	Tofacitinib (*n* = 2)	Azathioprine (*n* = 1)	Infliximab (*n* = 1)	None	8 ± 4.2	Clinical improvement (*n* = 2)	None
Régnier et al., 2019 [37]	3	Female (*n* = 2), male (*n* = 1)	40 ± 10	Baricitinib (*n* = 2), ruxolitinib (*n* = 1)	Methotrexate (*n* = 1), mycophenolate mofetil (*n* = 2)	Tocilizumab (*n* = 2), TNF inhibitors (unspecified) (*n* = 2)	No data	No data (*n* = 3)	Clinical improvement (*n* = 3)	None
Li et al., 2020 [38]	5	Female (*n* = 5)	22 ± 4.6	Tofacitinib (*n* = 5)	Methotrexate (*n* = 4), cyclosporine (*n* = 2), azathioprine (*n* = 2), mycophenolate mofetil (*n* = 4), leflunomide (*n* = 2)	Tocilizumab (*n* = 3)	No data	6–18	Clinical improvement (*n* = 4), no improvement (*n* = 1)	None
Kong et al., 2022 [39]	27	Female (*n* = 22), male (*n* = 5)	31.1 ± 9.2	Tofacitinib (*n* = 27)	No data (*n* = 27)	No data (*n* = 27)	None	12	Clinical improvement (*n* = 23), no improvement (*n* = 4)	Herpes zoster (*n* = 1)
Prakashini et al. 2023 [40]	10	Female (*n* = 9), male (*n* = 1)	28.3 ± 9.3	Tofacitinib (*n* = 10)	Methotrexato (*n* = 9), azathioprine (*n* = 2), mycophenolate mofetil (*n* = 9)	Etanercept (*n* = 1)	No data	6	Clinical improvement (*n* = 8), no improvement (*n* = 2)	None
Wang J et al., 2022 [41]	32	Female (*n* = 26), male (*n* = 6)	30.9 ± 9.0	Tofacitinib (*n* = 32)	No data (*n* = 32)	No data (*n* = 32)	None	12	Clinical improvement (*n* = 23), no improvement (*n* = 9)	Herpes zoster infection (*n* = 2), increase in the lipid level in blood (*n* = 1)
Zhou Z et al., 2024 [42]	10	Female (*n* = 9), male (*n* = 1)	29.7 ± 8.6	Baricitinib (*n* = 10) (one patient previously received tofacitinib)	Methotrexate (*n* = 5), leflunomide (*n* = 4), mycophenolate mofetil (*n* = 6), hydroxychloroquine (*n* = 4), tacrolimus (*n* = 2), sirolimus (*n* = 1), cyclophosphamide (*n* = 2)	TNF inhibitors (unspecified) (*n* = 3), secukinumab (*n* = 3), tocilizumab (*n* = 2)	Methotrexate (*n* = 7), leflunomide (*n* = 2), hydroxychloroquine (*n* = 3)	15.3 (range: 4–31)	Clinical improvement (*n* = 4), no improvement (*n* = 6)	Liver disfunction (*n* = 1)
Li J et al., 2025 [43]	10	Female (*n* = 9), male (*n* = 1)	29 [26–35.3]	Baricitinib (*n* = 10)	Methotrexate (*n* = 2), leflunomide (*n* = 1), azathioprine (*n* = 2), mycophenolate mofetil (*n* = 4), tacrolimus (*n* = 1), cyclophosphamide (*n* = 1)	Infliximab (*n* = 1), adalimumab (*n* = 6), etanercept (*n* = 3)	Methotrexate (*n* = 2), mycophenolate mofetil (*n* = 4), tacrolimus (*n* = 1), azathioprine (*n* = 2), leflunomide (*n* = 1), cyclophosphamide (*n* = 1)	11	Clinical improvement (*n* = 8), no improvement (*n* = 2)	Upper respiratory tract infection (*n* = 2), diarrhoea (*n* = 1)
Vasanth P et al., 2025 [44]	33	Female (*n* = 30), male (*n* = 3)	28.9 ± 7.6	Tofacitinib (*n* = 33)	Mycophenolate mofetil (*n* = 24), methotrexate (*n* = 20), azathioprine (*n* = 5), calcineurin inhibitors (*n* = 2), leflunomide (*n* = 1), cyclophosphamide (*n* = 2)	Tocilizumab (*n* = 14), anti-TNF (*n* = 14)	No data	15 [6.5–20]	Inactive disease (*n* = 23), no response (*n* = 5)	Myocardial infarction (*n* = 1), new onset QuantiFERON TB positivity (*n* = 1), herpes zoster (*n* = 1), transaminitis (*n* = 1)
Mekinian A et al., 2026 [45]	20	Female (*n* = 19), male (*n* = 1)	No data	Tofacitinib (*n* = 10), upadacitinib (*n* = 5), baricitinib (*n* = 4), ruxolitinib (*n* = 1)	Methotrexate (not specified number of patients), mycophenolate mofetil (not specified number of patients), azathioprine (not specified number of patients)	TNF inhibitors (not specified number of patients), tocilizumab (not specified number of patients), rituximab (not specified number of patients)	Methotrexate (*n* = 1), mycophenolate mofetil (*n* = 1), tocilizumab (*n* = 1)	Median time: 36 months	Clinical improvement (*n* = 10), no response (*n* = 5), relapse (*n* = 3)	Thrombosis (*n* = 1), infection (*n* = 1)
Current series	6	Female (*n* = 5), male (*n* = 1)	48.5 ± 9.6	Tofacitinib (*n* = 2), upadacitinib (*n* = 2), baricitinib (*n* = 2)	Methotrexate (*n* = 6), azathioprine (*n* = 1), mycophenolate mofetil (*n* = 2), cyclophosphamide (*n* = 1)	Etanercept (*n* = 1), infliximab (*n* = 4), tocilizumab (*n* = 4), rituximab (*n* = 1), ustekinumab (*n* = 1)	None	9.5 [6–16.7]	Clinical improvement (*n* = 4), no response (*n* = 2)	Tonsillar neoplasia (*n* = 1)

## Data Availability

The authors confirm that all data underlying the findings are fully available without restriction. All relevant data are included in the paper.
